# Supplementary figures and images for: Epigenetic and parasitological parameters are modulated in EBi3-/- mice infected with Schistosoma mansoni
Source: PLoS Negl Trop Dis. 2020 Feb 20;14(2):e0008080. doi: 10.1371/journal.pntd.0008080 (PMC7053770; doi:10.1371/journal.pntd.0008080)

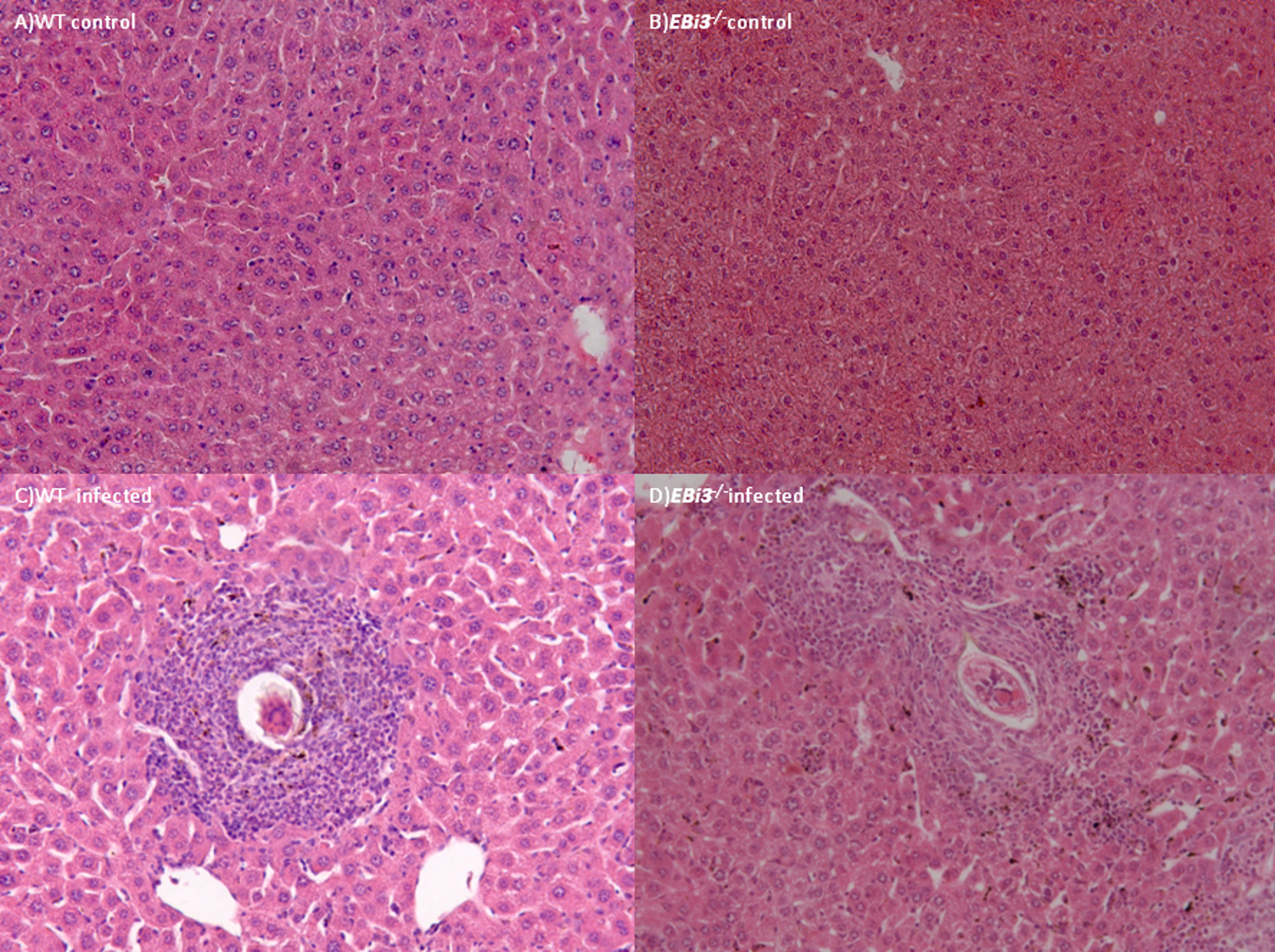

Supplement: S1 Fig — (A) Wild-type uninfected; (B) EBi3-/- uninfected; (C) wild-type infected; (D) EBi3-/- infected. Slides prepared after 55 days of infection; livers were fixed in 10%formaldehyde and stained using Hematoxylin eosin. (TIF) [file pntd.0008080.s001.tif]

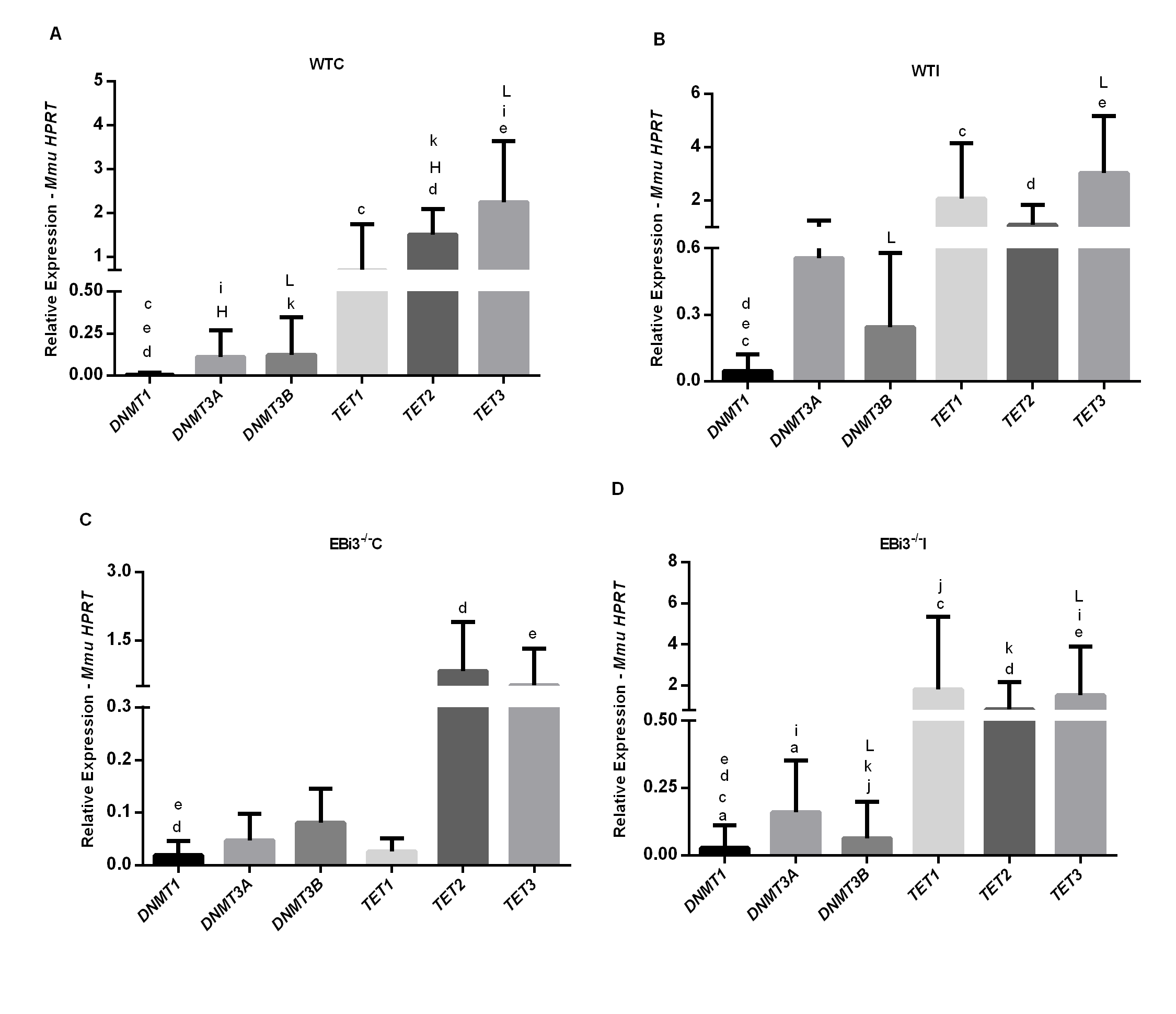

Supplement: S2 Fig — (A) WTC; (B) WTI; (C) EBi3-/-C; (D) EBi3-/-I. HPRT was used as constitutive gene control. Kruskal–Wallis, p < 0.05; GraphPad Prism6. Comparisons indicated by the following letters are significant. a: DNMT1 vs. DNMT3A; b: DNMT1 vs. DNMT3B; c: DNMT1 vs. TET1; d: DNMT1 vs. TET2; e: DNMT1 vs. TET3; f: DNMT3A vs. DNMT3B; g: DNMT3A vs. TET1; H: DNMT3A vs. TET2; i: DNMT3A vs. TET3; j: DNMT3B vs. TET1; k: DNMT3B vs. TET2; L: DNMT3B vs. TET3; m: TET1 vs. TET2; n: TET1 vs. TET3; TET2 vs. TET3. Error bars show the standard deviation. (TIF) [file pntd.0008080.s002.tif]

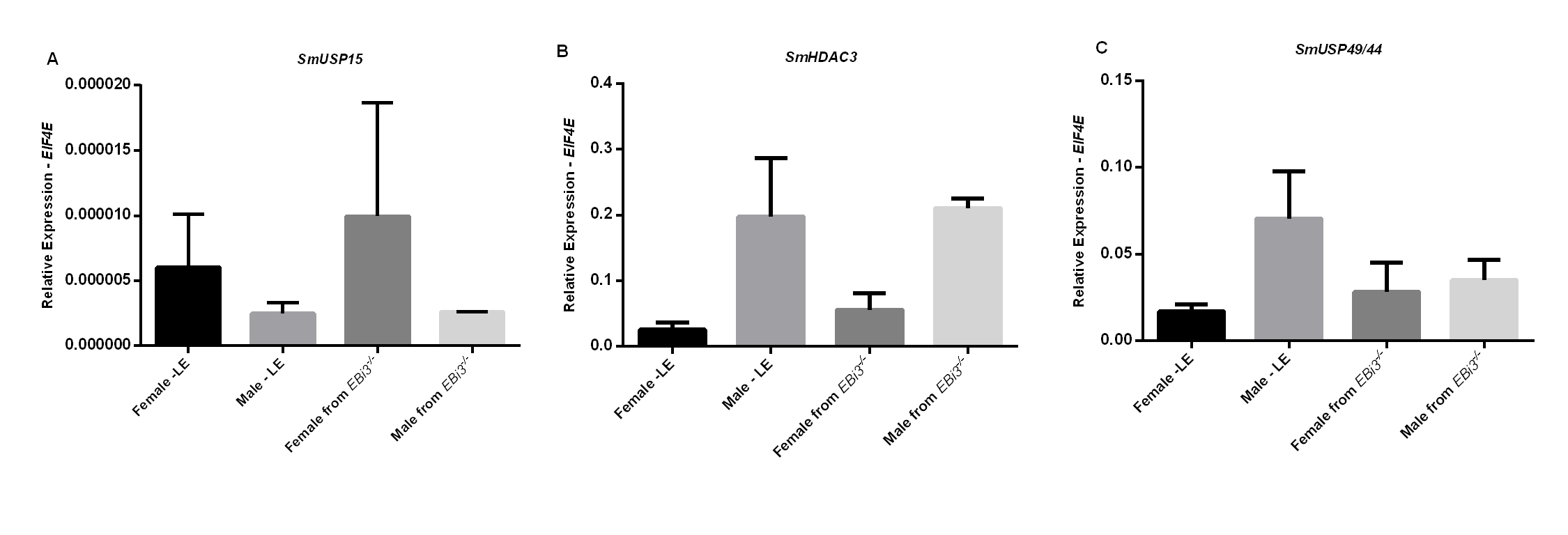

Supplement: S3 Fig — Female and male LE represent parasites recovered from WT mice; female and male EBi3-/- represent parasites recovered from knockout mice; all mice were infected with 100 cercariae. Experiments were done in triplicate using pools of at least 50 parasites per extraction. One-way ANOVA, p < 0.01; GraphPad Prism 6. No alterations in the expression levels of these genes were observed among the groups under our experimental conditions. Error bars show the standard deviation. (TIF) [file pntd.0008080.s003.tif]
