# Supplementary material for: Epigenetic and parasitological parameters are modulated in EBi3-/- mice infected with Schistosoma mansoni
Source: PLoS Negl Trop Dis. 2020 Feb 20;14(2):e0008080. doi: 10.1371/journal.pntd.0008080 (PMC7053770; doi:10.1371/journal.pntd.0008080)
Supplement: Attachment — Submitted filename: Response to Reviwers.docx [file pntd.0008080.s005.docx]

PNTD-D-19-01459

**Recommendation**

**Consider after major changes**

**Comments for the author**

We appreciate the reviewer comments and believe that we have addressed them properly. Every criticism was taken in a positive way. We revised the manuscript point by point and believe that we were able to answer all the questions and comply with the suggestions.

Clarifications required:

Reviewer #1: The hypothesis is stated but should be made clearer.

Answer: The hypothesis was changed.

Before: We hypothesize that *S. mansoni* alters its genomic imprinting in response to the immune system of the mammalian host, in this case a mouse knockout model, reprogramming gene expression and altering the number of eggs produced. In response, *S. mansoni* induces changes in the DNA methylation profile of hepatocytes that alter expression levels of genes modulating the extent of hepatic granuloma.

Now: We hypothesize that *S. mansoni* reprogram gene expression in response to the immune system of the mammalian host, in this case C57BL/6 *EBi3^-/-^* and alters the number of eggs produced. In response, mammalian host alters DNA methylation profile of hepatocytes by changing expression levels of *DNMT*s, *TET*s and the percentage of 5-mC and also modulates the extent of hepatic granuloma (lines 150-154).

Reviewer #2: The study design to address the stated objectives is poorly described. In particular the number of control mice (WTC, *EBi3^-/-^*C) and the number of infected mice (WTI and *EBi3^-/-^*I) used in this study is not specified, as well as the sex and age of the animals. A single infection experiment was performed in mice?

Figure 8:

**Fig. 8. Experimental scheme to compare molecular and parasitological parameters in mouse *EBi3^-/-^* and WT infected with *S. mansoni* and their influence on the male and female parasites.**

Male mice *EBi3^-/-^* and WT had 17 g and 5 weeks of life when they were infected and remained 55 days infected, and the uninfected groups *EBi3^-/-^*C and WTC waited the same time in the same conditions. During the euthanasia, feces of the last day were collected from the cages. The couple worms collected were mechanically separated by sex, counted and stored; the single worms were counted and stored too. Intestines weighting and identification allow collect data individually. The division of livers into four parts, identification of each mouse part and weighting in order to obtain data more precise.

Answer: Two euthanasias in different times were performed in mice. The number of control mice was 12 WTC and 5 *EBi3^-/-^*C, and the number of infected mice was 10 WTI and 15 *EBi3^-/-^*I for the first euthanasia. The second euthanasia was performed only with knockout mice 5 *EBi3^-/-^*C and 13 *EBi3^-/-^*I. A detailed description was made in Methods section (lines 599-618) and a figure 8 showing the experimental scheme. The mice were infected with 17 g and 5 weeks old, all males.

Reviewer #2: The RNA for RT-qPCR studies is prepared from pooled samples. It is not specified the number of mice used for the preparation of RNA. Regarding the correlation studies the statistical significance of the data should be also indicated in the Figure Legends.

Answer: The RNA for RT-qPCR studies in worms was prepared from pooled samples. The RNA of mice was prepared individually, as now described in Methods section (lines 674,675). The RT-qPCR studies contain all mice euthanized, 12 WTC, 10 WTI, 10 *EBi3^-/-^*C and 28 *EBi3^-/-^*I (2 euthanasias). The significance used for correlation is now specified in figure legend as well as the significance obtained. The significance of each graph is now indicated in the figure legends and also in Statistical Analysis in Methods section (lines 726-768). We made a section describing each statistical analysis used, p-value, p obtained, post-hoc test (if used) within Methods section.

Reviewer #1: -Does the analysis presented match the analysis plan?
Yes, but the results are difficult to follow.

-Are the results clearly and completely presented?

There are parts missing and presentation should be made clearer.

-Are the figures (Tables, Images) of sufficient quality for clarity?

Must be improved.

Answer: To improve the understanding and help track results we inserted a paragraph explaining the reason of those experiments in hypothesis tested, (lines 170-172, 209-212, 283-285, 289-292, 302-304, 364-366). Each figure legend in Results section was completed with statistical test details, as p-value, number of mice used to that experiment. Correlation matrix results were expanded to highlight the items that presented significance (lines 302-327). The miRNAs results from host and parasite were separated in different paragraphs to discriminate them and a little introduction of this result was inserted in the beginning to clarify the importance of them in schistosomiasis. The results of *USP*s expression and other epigenetic genes in *S. mansoni* were well described too (lines 381-406). Figure 7 was changed to highlight the results more important found in our analysis, Figure 8 was created to better understanding of experimental flow chart. All figures were adjusted in PACE as suggested by editor.

Reviewer #2: The text of the Results section is poorly presented. It could be helpful for readers to be guided in the list of genes that have been studied: i.e. criteria of selection, relevance. A more focused presentation on the most relevant gene expression variations could improve the quality of the manuscript. Maybe some data could be moved to the Supplemental data section.

Answer: in the Introduction section we inserted the function, and reason of choice of all genes analyzed, focusing in their epigenetic functions in mouse (lines 90-108) and in *S. mansoni* (lines 122-149). We expanded the matrix correlation description to improve the readers understanding. A short introduction to clarify the intention of each result was inserted after the subsection title. The degree of variation in % 5-mC of liver DNA and of *SmUSP*s expression was added as asked (lines 268-271 and 381-406). The genes that did not present statistical differences were in supplemental material.

Specific comments:

1. The data presented in Figure 1 are very interesting. The following information are not indicated: the numbers of control and infected mice used for the assessment of parasitological parameters; the number of independent infection experiments eventually performed; the sex and age of the mice used. These informations should be included in Figure Legend and in the Experimental section.

Answer: Parasitological parameters were obtained from the first euthanasia, 12 WTC, 10 WTI, 5 *EBi3^-/-^*C and 15 *EBi3^-/-^*I mice. These information are in figure legend (1), in Methods section (lines 603-618), and also in the figure 8 and figure legend (8). Figure 8 was made to clarify the methodology and the sample number used in each experiment. . The mice were infected with 17 g and 5 weeks old, all males.

2. Changes in de novo DNA methyltransferase and DNA demethylase expression in *EBi3^-/-^* linked to *S. mansoni* infection. Please specify that the analysis has been made on liver in the title. The significance is Kruskal–Wallis, p < 0.05 for all letters indicated? What each point represents?

Please indicate in the caption with kind of significance in the correlation has been observed. Spearman correlations: please describe better the parameters that you are correlating: granuloma volume as stated in legend or granuloma number as indicated in (G) and (H). Please specify in Figure 2 caption, which value of significance in the correlation has been detected.

Answer: You are correct; the significance is Kruskal–Wallis, p < 0.05 for all letters corresponding RT-qPCR. We inserted the statement in the figure legend (2): “Dots in all graphs represent a mouse, totalizing 60 mice of all groups” (lines 247-248). We correlated the two parameters, but only granuloma number was significant related to gene expression of DNMT1 and TET3. Now we inserted the statement in the figure legend: “Spearman correlations: gene expression level for each *EBi3^-/-^*I mouse was correlated with granuloma number for the same mouse, all correlations were tested using p < 0.05” (lines 244-245). In the figure legend (2) we inserted the r value, both graphs correlated negatively (line 245-246).

3. Please indicate in each Figure legend what each point represents (sample/mouse/technical replicate/biological replicate).

Answer: Each dot represents a mouse, and now we specified it in the figure legends of figure 2 and figure 4 (lines 247, 248 and 333, 334).

Reviewer #1: -Are the conclusions supported by the data presented?

Yes. But presentation and logics could be improved.

-Are the limitations of analysis clearly described?

No.

Answer: The limitations of lncRNAs expression have been included in lines 350-355. The conclusions are in the 2 last paragraphs of discussion (lines 547-581).

Reviewer #2: The manuscript lack Conclusions and the authors do not discuss how these data can be used for further investigation on the role of the host immune system on schistosomiasis.

Answer: The discussion involving host immune system was added in lines 547-566.

“Decreased oviposition and worm size has already been observed in other models of knockout, such as hepatic CD4 ^+^ T-lymphocyte^-/-^, in addition to decreased male-female pairing [44]. More recent studies have detected that nematodes are stimulated by the induction of some interleukins. The presence of IL 5 increased nematode reproduction and parasites recovered from IL 5^-/-^ mice had larvae size smaller than the larvae size recovered from control, thus concluding that this interleukin promoted faster development and reproduction without altering other parasite characteristics [45]. Our EBi3 knockout model may have induced decreased worm oviposition. It has been described that p28 and p35 generate signaling independent of EBi3 protein, but this signaling has unknown function and impact so far [7]. In the study that assessed the impact of the WSX-1 knockout chain (the only receptor chain that is not shared with other interleukins besides IL27) the authors detected a decrease in IFNɣ [6]. In this study, the absence of IL27 due to EBi3 knockout could also have triggered a decrease in IFNɣ and a decrease in granulomas volume, since this cytokine is important in the acute phase of schistosomiasis. The impact of IL35 should be more evident in the chronic phase of schistosomiasis mansoni, in which there is an increase in the regulatory immune response, since IL35 has a strong anti-inflammatory/regulatory function. The host reacted to this softer pattern of infection and modulated the expression of genes responsible for alterations in silencing and activation in DNA, which may interfere with the transcriptional regulation of several genes (Fig 7)”.

Reviewer #1: Mota et al. describe a very interesting phenomenon and the subject is definitely worth publishing. However, the matter of mutual influences of epigenetic information is complex and the manuscript was in this regard a bit difficult to follow. Essentially, I was often lost in my understanding if the authors talk about Schistosoma or mouse gene expression, RNA presence, modifications of DNA methylation etc.
Please make sure that the reader always knows where he is (in the parasite or in the host) and what the origin of the molecular is (parasite or host). This applies also to the discussion. Please do an effort to make clear where we are and who we talk about, and differentiate it also from the general case/assumption (e.g. line 367 I suppose it is meant that “in general HDAC8 shows nuclear and cytoplasmic location...”
DNA methylation is used equivalent to 5-methyl-cytosine, but there are more methylation modifications than this. Make this clear from the beginning or change terminology.

Answer: In relation to the presentation of data we effort to introduce the target (i.e host or parasite) of each analysis in the beginning of description. We modified the description of HDAC8 and moved it to Introduction section. We also specified 5-methyl-cytosine in the descriptions of ELISA results.

I find “epigene” a bit strange and it took me a while to understand that genes coding for chromatin modifying enzymes and chromatin associated RNA is meant.

Answer: This term was changed to “Genes modulators of epigenetics”, lines 408 and 649.

I recommend that these general points and the specific points below should be addressed by the authors before further review.

Specific comments

Line 19: “genomic imprinting” is a reserved term for imprinted genes. Don’t use it in your context.

Answer: This term was changed to epigenetic state, line 19.

Line 21: “could occur” since it’s a hypothesis

Answer: “could occurs” was inserted, line 21.

In abstract and author summary explain what epigenetics (and stick to this definition throughout the manuscript) is and why it was investigated. Develop in more detail in the introduction.

Answer: “Epigenetics comprises changes in chromatin that do not involve changes in nucleotide sequence. These changes are triggered by environmental factors such as diet, social interaction, physical exercise, disease contagion, age, among others. Among the epigenetic alterations we can mention modifications in the N-terminal portion of the histone tail (acetylation, deacetylation, ubiquitination, deubiquitination, methylation, demethylation, among others), DNA modifications, such as methylation and demethylation of cytosines, regulation by microRNAs and Long non-coding RNAs. These changes are fundamental for gene expression regulation, sex X chromosome silencing in females, transposon silencing [8,9] (lines 90-98)”.

Line 82: not clear why there is a link between sentence finishing by “...adaptation to the host.” and the next one “Among other factors...”

Answer: “Among other factors” was removed from the text, line 111.

Line 87: “insert” should read “influence”?

Answer: “Insert” was changed to trigger into host new…, line 116.

Line 103: don’t use “imprinting”

Answer: The hypothesis was changed and this word was not included, lines 150-154.

Line 104-105: don’t understand the sentence

Answer: The hypothesis sentence was changed, lines 150-154.

Line 108: mention here which are the parasitic parameters that change

Answer: The parasitic parameters changed are now described in lines 155-157.

*Number of eggs per gram of liver, number of hepatic granulomas, granuloma volume and number of eggs per gram of feces.

Line 149: in which tissue?

Answer: The tissue used was the liver, line 208

Line 180: talking about demethylating enzyme activity or 5mC level?

Answer: “Considering the relative expression levels of TET and DNMT genes” was included in this position, line 251.

Line 196: 0.5% of what?

Answer: 0.5% of 5-mC was included in the text, line 266.

Line 194-202: good to know that the increase is statistically significant but what is the extent? Fold change or % difference with SD

Answer: *“EBi3^-/-^*C had 1.58 times more of % 5-mC than WTC; this difference was lost (*EBi3^-/-^*I vs. WTI, p < 0.05). *S. mansoni* infection reduced % 5-mC of hepatic DNA rates significantly in *EBi3^-/-^*I mice (p<0.05) but not in WTI mice (Fig 3), *EBi3^-/-^*C mice contained 1.96 times more of % 5-mC than *EBi3^-/-^*I” (lines 269-272).

The text above was included as requested to elucidate the kind of difference.

Line 292: should this read “BUT induction of Th2...”?

Answer: “But” was included in line 427.

Line 302: should “due to” read “judging from” or similar?

Answer: “judging from” included in line 437.

Line 317-330: I don’t get the point the authors want to put forward here.

Answer: the paragraph was completely changed, lines 451-469.

“The DNA methylation and demethylation of liver affected by schistosomiasis remains unexplored. In our approach the S2 highlights the expression of all genes in each group facilitating the perception of patterns, for example, analyzing each group separately we can see tendencies to methylation of hepatic DNA during infection considering WTC versus WTI. *S. mansoni* infection led to an increase in DNA methylation in WTI mice, indicated by a decrease of statistical differences among relative expression levels of all *TET* and *DNMT* genes in the WTI group compared to statistical differences among *TET* and *DNMT* expression levels in WTC; comparing Fig 2 with S2 suggests that *DNMT3A* plays an important role in the rate changes observed. In *EBi3^-/-^* mice, infection caused an increase in DNA demethylation and analyzing *EBi3^-/-^*C versus *EBi3^-/-^*I we can see that *TET1* plays the major differences observed in S2, but *TET2* and *TET3* also increased strongly their expression values. The literature lacks data about methylation and demethylation of DNA in hepatic tissues during schistosomiasis, but considering other disease that affect the liver causing collagen deposition, there is an increase in liver methylation during hepatic fibrosis development, as determined by analyzing the overall methylation and the methylation of specific genes (Septina9, Long non-coding RNA H19, SUN2) in diseases such as hepatocarcinoma, hepatitis and cirrhosis [10,11,28], this occurred in our control groups WTC and WTI considering the qRT-PCR data.”

Line 377: should read “…for the methylation and demethylation in the host (?),…” but then why methylation AND demethylation?

Answer: The paragraph of conclusions was modified (lines 547-581).

Line 380: the figure is not well drawn and as confusing as some parts of the manuscript. It is not clear what is host and what is parasite. Maybe color code this and make the figure legend more clear.

Answer: Figure 7 was modified, see the archive.

**Fig. 7. Effect of the mouse *EBi3^-/-^* immune system on schistosomiasis.**

Comparative scheme of the main immune system impacts on parasite and host. The immune system can modulate gene and miRNA expression in worms and the quantity of eggs they release resulting in less hepatic damage. In definitive host the number of granulomas, eggs/g of feces, expression of *TET3* were reduced, besides the increasing the % 5-mC in liver DNA.

Reviewer #2: The authors used C57BL/6 and C57BL/6 *EBi3^-/-^* murine (*Mus musculus*) models of *S. mansoni* infection in order to investigate possible changes in new and maintenance DNA methylation profiles in the liver after 55 days of infection. Moreover, the authors evaluated the expression levels of epigenetic genes and genes linked to histone deubiquitination in male and female *S. mansoni* worms by RT-qPCR. The subject is novel and of significance in the Schistosomiasis field and in particular in the host-parasite interplay. The data are poorly presented but the Manuscript can be improved. The most relevant findings should be highlighted. Importantly, the criteria used for the selection of genes to be investigated are not clearly presented. In the introduction an overview of the genes investigated in the paper should be included. Some information that is in the discussion section should be in the introduction to better follow the data presented in the Results section. The paper lack any Conclusion.

Answer: *S. mansoni* genes involved in epigenetics were well described in Introduction (definition and criteria, lines 122-149) and Results sections (lines 381-406). *M. musculus* genes involved in epigenetics were also described in Introduction section (lines 90-108). Correlation matrix was well described in lines 302-327. MicroRNA Results and Discussion section were improved too (lines 470-504). Conclusions paragraph were inserted (lines 547-581).
